# Evaluation of *Dakshata*, a scale-up WHO SCC and mentoring-based program, for improving quality of intrapartum care in public sector in Rajasthan, India: repeated mixed-methods surveys

**DOI:** 10.1186/s13690-023-01028-z

**Published:** 2023-04-18

**Authors:** Samiksha Singh, Nanda Kishore Kannuri, Aparajita Mishra, Leena Gaikwad, Rajan Shukla, Mukta Tyagi, Swecha Chamarty

**Affiliations:** 1 Indian Institute of Public Health-Delhi, Public Health Foundation of India, Delhi, India; 2 Indian Institute of Public Health-Hyderabad, Public Health Foundation of India, Hyderabad, India

**Keywords:** WHO safe childbirth checklist, Checklist, Mentoring, Quality improvement, Evidence-based practices, Childbirth, stillbirth, India

## Abstract

**Background:**

The *Dakshata* program in India aims to improve resources, providers’ competence, and accountability in labour wards of public sector secondary care hospitals. *Dakshata* is based on the WHO Safe Childbirth Checklist coupled with continuous mentoring. In Rajasthan state, an external technical partner trained, mentored and periodically assessed performance; identified local problems, supported solutions and assisted the state in monitoring implementation. We evaluated effectiveness and factors contributing to success and sustainability.

**Methods:**

Using three repeated mixed-methods surveys over an 18-month period, we assessed 24 hospitals that were at different stages of program implementation at evaluation initiation: Group 1, training had started and Group 2, one round of mentoring was complete. Data on recommended evidence-based practices in labour and postnatal wards and in-facility outcomes were collected by directly observing obstetric assessments and childbirth, extracting information from case sheets and registers, and interviewing postnatal women. A theory-driven qualitative assessment covered key domains of efficiency, effectiveness, institutionalization, accountability, sustainability, and scalability. It included in-depth interviews with administrators, mentors, obstetric staff, and officers/mentors from the external partner.

**Results:**

Overall, average adherence to evidence-based practices improved: Group 1, 55 to 72%; and Group 2, 69 to 79%, (for both *p* < 0.001) from baseline to endline. Significant improvement was noted in several practices in the two groups during admission, childbirth, and within 1 hour of birth but less in postpartum pre-discharge care. We noted a dip in several evidence-based practices in 2nd assessment, but they improved later. The stillbirth rate was reduced: Group 1: 1.5/1000 to 0.2; and Group 2: 2.5 to 1.1 (*p* < 0.001). In-depth interviews revealed that mentoring with periodic assessments was highly acceptable, efficient means of capacity building, and ensured continuity in skills upgradation. Nurses felt empowered, however, the involvement of doctors was low. The state health administration was highly committed and involved in program management; hospital administration supported the program. The competence, consistency, and support from the technical partner were highly appreciated by the service providers.

**Conclusion:**

The *Dakshata* program was successful in improving resources and competencies around childbirth. The states with low capacities will require intensive external support for a head start.

**Supplementary Information:**

The online version contains supplementary material available at 10.1186/s13690-023-01028-z.

## Background

High quality care during childbirth is essential for better maternal and newborn outcomes [1, 2]. Although there is substantial evidence of clinical practices positively affecting healthoutcomes, practices are often missed due to a lack of knowledge, skills, resources, comprehensive protocols, monitoring or accountability [2–4]. The WHO Safe Childbirth Checklist (SCC) [5] identified crucial moments where a set of evidence-based practices should be performed to ensure high quality services. The Checklist can be used as a reminder tool or job aide, as well as a monitoring tool. The Checklist has been pilot tested and implemented in several countries using a variety of strategies, including training about the use of checklists, additional knowledge and skills training on the clinical practices, coaching and quality improvement approaches [6–13].

In India, the Checklist was pilot tested to assess the acceptability and evaluate change in provider behaviour in the states of Rajasthan, Karnataka, and Uttar Pradesh [7, 9, 10, 14]. The BetterBirth project in Uttar Pradesh built in coaching along with the use of SCC and showed improvement in adherence practices but did not show changes in maternal or newborn mortality [7, 8, 13]. The SCC pilot in 7 districts of Rajasthan (2012–15) demonstrated a decrease in early neonatal mortality [13], and was thus scaled-up into a national program—the *Dakshata* program [15] where the WHO SCC is adopted into a 29-item facility-based training tool coupled with mentoring. Additionally, the *Dakshata program* incorporated improving resources and program monitoring led by the state [15].

India has more than 80% hospital deliveries, and a slow declining maternal mortality ratio (MMR) currently at 103 per 100,000 [16] and a stillbirth rate of 4 per 1000 live births [17]. There was a noted improvement in the proportion of institutional deliveries over pasta decade [18, 19], but the quality of childbirth services and referrals did not improve correspondingly [20–22]. In recent years, the Government of India has emphasized strengthening infrastructure and quality of obstetric care through several programs [15, 23–25]. Amidst all these efforts, there is a need to assess the effect of the *Dakshata* program, which is one of the first comprehensive quality improvement programs for maternal/newborn care in the country.

### Intervention- *Dakshata* program in Rajasthan

The *Dakshata* quality improvement program was planned to be implemented in government-run secondary and primary care hospitals. The JHPIEGO-India office supported the Rajasthan state in scaling-up the program to 20 districts (excluding the 7 pilot districts) in a phased manner, covering over 200 hospitals between 2015 and 2019. The program districts and hospitals were jointly selected by the respective state health departments and the JHPIEGO state consultants; the evaluation team did not have any role in this selection or implementation.

The *Dakshata* program aimed to improve providers’ competence and accountability, while ensuring appropriate resources in labour rooms and wards. The intervention package included: “a) bulk training of 3 days for use of SCC for essential practices in the labour room, b) mentoring and support visit (MSV) package of 3-4 months of on-site pulse training, c) technical support required to ensure availability of resources, and d) technical support to state and country for strategic planning, and monitoring” [15]. *Dakshata’s* skill building focused on practices from four crucial pause points as in WHO-SCC [5]: Pause point 1 at the time of admission; Pause point 2 just before and during childbirth; Pause point 3 immediately after childbirth (within 1 hour); and Pause point 4 at the time of discharge [5, 15]. Rajasthan government incorporated the SCC in standardised case sheets (medical records) for the labour rooms in the public sector.

JHPIEGO provided intensive support to state through a team of 3–4 state and 10 district program officers. The JHPIEGO state program officers provided 5 days of training to a pool of handpicked government trainers from each program district, regarding clinical as well as pedagogical skills. These government trainers, under the supervision of JHPIEGO program officers, trained the staff (Medical Officers, Staff Nurses, and Auxiliary Nursing Midwives (ANMs)) from the target hospitals in their districts, for 3 days as per the established labour room protocols and standards. The training was provided at the District hospitals in small batches to facilitate training of at least 80% of the eligible staff without disturbing the hospital-level functioning. Post-training, the JHPIEGO program officers (designated *Dakshata* mentors) provided mentoring through a structured 8-visit package (consolidated to 5 visits in late 2018). Mock-drills and feedback in real settings and repeat training were the standard mentoring approaches. The mentors also got involved in problem-solving and team building at the hospitals. The mentors conducted quarterly periodic assessments using a structured format to monitor the progress in the availability of supplies, adherence to essential practices, and record keeping. They provided feedback to hospitals, districts, and states. Table 1 describes the implementation components in further detail. Typically, in a district, it took 4–6 months to complete 3-day training for the eligible staff; 6–8 months to complete all 8 mentoring visits (4–5 months for 5 mentoring visits) per facility, and more than 3 months of need-based mentoring for lagging practices till planned handover to state.

**Table 1:** Summary description of intervention in Rajasthan as of January 2019

Mentoring and Support Model: External mentors mentored staff using a standard package	
**Intervention hospitals**	Over 200, including District hospitals, Sub-district hospitals, and high delivery load Community Health Centres and Primary Health Centres.
**Support from technical partner**	They conducted rapid assessments of infrastructure and resources and suggested improvements for better preparedness before training. They trained Government identified *Dakshata* trainers in each district and supervised the training of the obstetric staff. They helped in micro-planning of training and facilitated the operations as per the rosters. They mentored and conducted periodic assessments at the facility level; regularly provided feedback to hospitals, district, and state administration.
**Bulk training**	Training included the use of SCC, and the advancement of clinical knowledge and skills for risk assessment, the conduct of vaginal delivery, infection control, and management of common complications of the mother, birth asphyxia, and newborn hypothermia. It also covered referral criteria and pre-referral stabilizing care. The trainers conducted Pre- and Post- OSCIs to measure the effect of training and provided feedback to the trainees.
**Pulse mentoring**	Mentoring consisted of pulse training, mock-drills, and feedback for corrections; also consultation to ensure the availability of essential resources. Mentors used a structured mentoring package (8 visits later consolidated to 5). Once this package was completed, mentors continued to provide need-based mentoring based on the specific gaps identified from periodic assessments.
**Periodic assessments**	They scored the performance (using a 19-point composite score), identified gaps, provided feedback, and implemented corrections. The mentors used a mobile-based software application that provided a summary report in real-time which was also available to the program managers in the district and state.
**Monitoring and supervision by state**	The team from JHPIEGO monitored and provided feedback to the state and district department. The Government maternal health officers in state directly monitored the mentoring and performance of the facilities. They acknowledged and rewarded the better performing labour room teams, and also provided support where required.
**Phase out**	Phase-out was planned for October 2019. During phase-out and handover to the state, the government recruited 17 mentors who interned under JHPIEGO mentors for 2 months, before taking up complete handover. Additionally, the state identified block (sub-district) mentors from the in-service obstetric teams, who were trained by JHPIEGO mentors and were to provide clinical support and mentoring to 2–3 facilities in their region. These mentors were expected to be self-sufficient. The state and district administration acquired management, supervision, and monitoring responsibilities.

The 20 districts were covered in 2 phases: phase 1 initiated in 2015–16 in 13 districts and phase 2 initiated in 2017–18 for 7 districts, i.e. 20 in total. At the time evaluation was planned, all the program districts had received/just initiated the intervention, and were at different stages of implementation. For this evaluation, we defined four stages of implementation; 1) no intervention started, 2) 3 days of training completed, 3) all 8 or 5 mentoring package visits completed, and 4) at least three need-based mentoring visits completed, and the handover was planned. The progress of the program implementation between January 2016 and January 2020 is presented in Table 2. The stages were used for stratified sampling, planning evaluation follow-up intervals, and appropriate data interpretations. This *Dakshata* program evaluation was conducted to 1) evaluate the effect on adherence to maternal and newborn health evidence-based provider practices, and stillbirths; 2) assess the efficiency of the program components, monitoring, and accountability to improvement in the quality of services; and 3) assess the adequacy of external support, institutionalisation in government systems, sustainability and scalability of the program.

**Table 2:** Status of the Dakshata program in Rajasthan between 2015 and 2020

State and stages of intervention, ***N*** = no. of districts	2012–2015	January 2016 ***N*** = 20	January 2017 ***N*** = 20	January 2018 ***N*** = 23	January 2019 ***N*** = 30	January 2020
**Intervention not started**	Pilot in 7* districts (*not included in current evaluation)*	17	7	0 + 3**	0 + 7*	Government mentors lead mentoring in all 30 facilities; JHPIEGO managers provided regional support
**Initial 3-day (Bulk) training completed/near completion**		3	3	6	0	
**Mentoring package completed**		0	7	11	6 + 3**	
**Need-based mentoring completed / near phase-out)**		0	3	3	14	

*7 pilot districts and **3 additional districts added on request of the state with light support by JHPIEGO

## Methods

### Study design

**Fig. 1:**
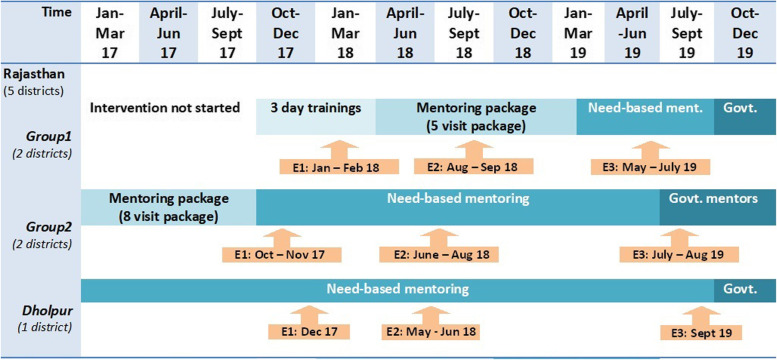
Timelines for stages of intervention and repeated assessments in the study hospitals in Rajasthan. Legend: Quantitative data from Dholpur is not included in the analysis but qualitative analysed and is presented in the results

Using a quasi-experimental study design, we conducted 3 repeated mixed-methods cross-sectional surveys without controls. The first assessment was conducted between October 2017 and February 2018, the second assessment between May and September 2018, and the third assessment between May and September 2019 (Fig. 1). Feedback and discussions with stakeholders through March 2020 were also incorporated into the qualitative findings and discussion.

### Study population

It considered of pregnant women who delivered and their newborns and health staff (doctors and nurses) in the labour rooms of secondary public health hospitals assigned for the *Dakshata* program. We interviewed JHPIEGO program managers and mentors, and hospital, district and state government administrators responsible for maternal health programs.

### Outcome indicators

The evaluation team identified 5 measurable indicators per pause point, i.e. 20 in total, to assess high quality care. The indicators pertaining to the actual management of the complications, although suggested, could not be used as the number of women with complications was small and the case sheets did not provide sufficient detail due to incomplete or improper documentation during the pilot. The in-facility outcomes included the caesarean section rate, stillbirth rate, and referral of mother and newborn.

### Sample size and sampling

**Fig. 2:**
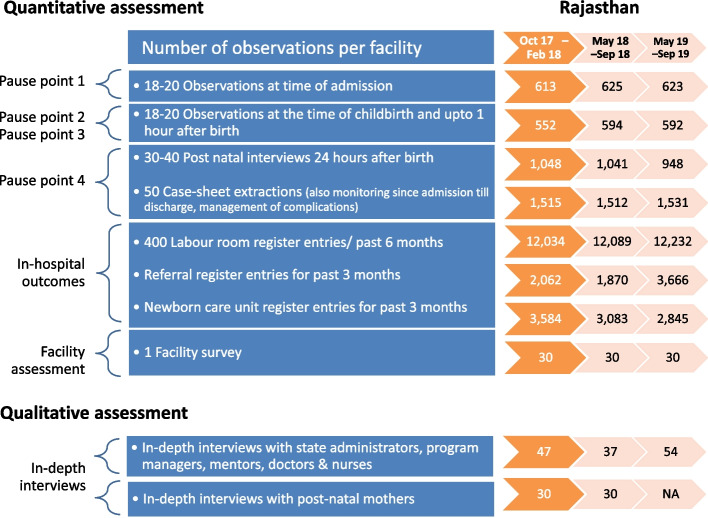
Sample included from 30 facilities during the three assessments in Rajasthan. Legend: We observed 2–3 additional admissions or childbirths per facility to adjust for overall sample size, in case we had fewer numbers from any facility or if we had to discard any case during data cleaning. One facility did not have/ had very less vaginal deliveries within 10 days of our observation in the first assessment. Among the facilities; 13 in time 1, 11 in time 2 and 3 did not have NBSU/SNCU. Quantitative data from Dholpur is not included in the analysis but qualitative analysis is presented in the results

We estimated the sample size for repeated cross-sectional assessments for cluster (hospital) sampling [26]. (Fig. 2) We used the percentage adherence to identified evidence-based practices from the baseline of program data [10] and computed the sample size for each to detect the desirable change (increase to 80% adherence from baseline) with 80% power and 95% confidence, intra-cluster correlation of 0.1 and cluster size 18 observations. The largest sample size was 30 facilities × 18 observations for a total of 540 observations. For less prevalent practices regarding complications, post-natal care, and in-facility outcomes, we planned for a higher number (based on feasibility) i.e. 50 case sheets, 30 post-natal mother interviews, and 400 labour room register entries per hospital. We randomly sampled 5 districts, stratified for the stage of the program implementation, region, and neonatal mortality rate. At the time of the first assessment, two sampled districts from Rajasthan (Barmer and Jaipur-1) were conducting three days of bulk training (Group 1); two districts (Udaipur and Nagaur) had just completed 8 visits mentoring package (Group 2), and the fifth district (Dholpur) was near phase out. Within each district, we selected six hospitals from within the intervention hospitals in consent with District health officers; these included District hospitals (2), Sub-district hospitals (5), Community health centres (15) and Primary health centres (2). At each repeated assessment, we consecutively included the required number of observations of admissions and childbirths, case sheets, register entries, and interview of post-natal women. For qualitative assessments, we purposively selected two districts based on the stage of program implementation: Dholpur had completed a few need-based visits; and Barmer was completing bulk training. We conducted extensive stakeholder analysis to identify the key personnel, and their roles, that could influence the program. We included all the eligible leaders, administrators, managers, mentors, and hospital in-charges in the sample frame. We interviewed a purposive sample of the available service providers and 5–6 beneficiaries per facility.

### Data collection

We directly observed (non-participatory) service provided to pregnant women at the time of admission, during, and immediately following childbirth in the labour room 24 hours a day consecutively till the sample size was met, and recorded information using observation checklists. We interviewed pregnant women in the post-natal and post-op wards using a structured interview guide. We extracted information from case-sheets post-discharge onto a data extraction checklist. If the number of discharges was short of 50 during our visit, we included remaining from prior days going backward. We conducted a facility survey to assess general infrastructure and resources and extracted information on childbirth outcomes from birth and referral registers in labour rooms or obstetric wards. Data were collected by qualified nurses who were trained intensively for 5 days, under the supervision of team leads. It took about 5 to 15 days to complete data collection per hospital. We pilot-tested all the quantitative data collection tools before use and developed them into an Android-based application for recording data; and built-in logic checks and restrictions to minimize wrong entries. We used Lenovo tablets for collecting data in the field and uploaded them after finishing data collection from each facility. Data were directly saved on the server from where the central research team extracted data on Stata 14.0.

We designed In-depth Interview (IDI) guides, covering key domains of efficiency, effectiveness, institutionalization, accountability, sustainability, and scalability. We developed guides for different cadres in English. We translated them into the local language (Hindi and Telugu) and back-translated to English, corrected errors and pilot tested them. We conducted interviews in the providers’ workplaces. The interviews with district and facility level stakeholders were conducted by two trained researchers under the guidance of the co-investigator, all of whom were social anthropologists in the health sector. Another two trained research staff conducted in-depth interviews of post-partum women. We audio recorded the interviews, if consent was provided, and took handwritten notes. The team transcribed and translated the recordings. Some of the respondents refused a formal interview so we only had informal discussions and noted important aspects of the program. We also took notes in the meetings with JHPIEGO or State government officials, that could be of significance for understanding the implementation process and strategies.

### Ethics

We obtained ethics approval from the Indian Institute of Public Health-Hyderabad. We obtained written informed consent before the interviews. As the observations did not require us to interact with the women giving birth, and we did not want to interfere with the routine process of care, thus we did not obtain beneficiary consent, but we did obtain permission from hospital superintendents and health staff.

### Analysis

We used STATA 14.0 for data management and analysis. For each clinical practice, we computed the proportion of adherence for a hospital (cluster) and then computed the average of these proportions weighted for monthly delivery load in the hospitals. We also computed average adherence to the five practices for each pause point, and overall average adherence for the 20 practices. We conducted linear regression for a testing average of proportions over time, and Poisons regressions for stillbirths and referrals. We scored the performance of hospitals and gave a score of one if the adherence to practice was 80% or more. A hospital could get a maximum score of 5 for a pause point, and 20 overall. A score of 4 or more in pause points and 14 or more overall was considered satisfactory. We conducted a qualitative analysis based on pre-defined themes and sub-themes, and also identified new emerging themes. We used Atlas-Ti to code the qualitative interviews and notes. We present integrated qualitative results from all three timepoints accounting for implementation progression and effects on the course.

## Results

We present results from only Group 1 and 2 sample hospitals, See Additional Table 1 for details. The program was not appropriately implemented in District Dholpur due to several administrative and local facility level determinants as mentioned by the district and state officers, thus we dropped it from the final quantitative analysis in consent with the state, and technical partner. However, there were health system learnings, so we retained it in the qualitative analysis.

The program in Group 1 progressed from bulk training during the first assessment to complete three need-based mentoring, while Group 2 completed one and half years of need-based mentoring and phased out by handover to Government mentors. Amongst the analysed 24 hospitals, the district hospitals noticed an increase in monthly deliveries, while the delivery load reduced in lower facilities, likely due to increased referrals. The availability of staff and caesarean services were consistent. The availability of 5 out of 6 assessed protocols improved, but the availability of protocols for antenatal corticosteroids and preterm labour were still limited-- found in only 12 and 9 of 24 hospitals, respectively. All amenities for maintaining hygiene improved over time in all hospitals. The availability of essential trays improved in Group 1 facilities to 10 or more, while it reduced in Group 2 to 8 or less, due to ongoing renovations in a few facilities. (Additional Table 1 ).

Adherence to evidence-based practices in obstetric care (Table 3).

**Table 3:** Adherence to evidence-based practices, per pause point and overall, in facilities under study, over time, % (95% C.I)

	Group 1, ***N*** = 12 facilities			Group 2, ***N*** = 12 facilities		
	***Bulk training ongoing***	***Mentoring ongoing***	***Need based mentoring ongoing***	***Mentoring completed***	***Need based mentoring ongoing***	***Need based mentoring for a year***
**Blood pressure measured (obs)**	52 (30–74)	63 (45–81)	**83 (78–89)**	67 (51–83)	61 (27–94)	**97 (95–99)**
**Foetal heart sounds assessed (obs)**	70 (59–81)	80 (64–96)	**84 (79–90)**	82 (75–89)	85 (77–92)	**96 (93–99)**
**PA examination (obs)**	86 (78–93)	83 (67–99)	**95 (90–100)**	91 (86–97)	65 (40–89)	96 (93–99)
**PV examination (obs)**	93 (88–98)	**100 (99–100)**	97 (95–100)	99 (98–100)	97 (95–99)	100
**Hand Hygiene in PV examination (obs)**	16 (3–29)	31 (4–58)	**73 (69–78)**	49 (40–58)	30 (5–55)	**90 (81–100)**
*Average Pause point 1*	*63 (53–73)*	*71 (61–82)*	***87 (83–90)***	*78 (73–82)*	*67 (52–83)*	***96 (94–98)***
**Pre-filled oxytocin (obs)**	67 (51–83)	70 (55–85)	**97 (95–99)**	78 (71–86)	81 (72–91)	**94 (87–100)**
**Ready bag and mask (obs)**	85 (72–98)	94 (88–100)	70 (51–89)	97 (94–100)	88 (70–100)	92 (84–100)
**Clean, dry and warm towels (obs)**	47 (25–69)	67 (42–92)	**76 (62–90)**	74 (55–93)	65 (53–76)	82 (72–92)
**Used clean cord cut (obs)**	99 (98–100)	100	99 (98–100)	99 (98–100)	99 (97–100)	100
**Oxytocin within 5 minutes (obs)**	74 (63–85)	85 (77–94)	77 (64–89)	83 (78–87)	84 (77–91)	**94 (91–97)**
*Average Pause point 2*	*74 (65–84)*	*83 (76–91)*	*84 (78–89)*	*86 (81–92)*	*83 (77–89)*	*92 (89–96)*
**Baby dried immediately (obs)**	86 (74–98)	**98 (96–100)**	84 (73–95)	99 (97–100)	94 (89–99)	90 (80–99)
**Baby weight observed (obs)**	71 (59–82)	86 (75–97)	79 (68–90)	96 (93–100)	75 (64–86)	64 (44–83)
**Breast feeding initiated within one hour (obs)**	42 (12–73)	54 (30–77)	**79 (69–89)**	45 (30–59)	48 (35–61)	**78 (69–88)**
**Assessed uterine tone (obs)**	73 (63–84)	30 (14–46)	72 (61–83)	82 (72–93)	38 (16–59)	69 (50–89)
**Mothers vitals checked (obs)**	13 (2–24)	16 (4–27)	**67 (56–77)**	25 (7–44)	9 (2–17)	**82 (67–97)**
*Average Pause point 3*	*57 (51–63)*	*57 (50–63)*	***76 (68–85)***	*69 (64–75)*	*53 (46–60)*	*76 (70–82)*
**Newborn immunised (cs)**	10 (4–16)	27 (0–55)	31 (0–65)	56 (34–78)	56 (34–78)	42 (11–74)
**Mother’s temperature measured (cs)**	46 (23–69)	56 (18–94)	64 (42–87)	61 (39–84)	61 (39–84)	**98 (95–100)**
**Counselled for any danger sign in newborn (int)**	17 (11–24)	15 (0–34)	11 (7–15)	21 (9–33)	19 (8–30)	3 (1–6)
**Counselled for any danger sign in mother (int)**	18 (10–26)	10 (0–21)	21 (12–30)	27 (4–50)	8 (3–14)	35 (0–75)
**Counselled for family planning (int)**	30 (13–48)	**60 (39–80)**	**71 (50–92)**	57 (38–77)	20 (6–33)	**74 (57–92)**
*Average Pause point 4*	*24 (18–30)*	*34 (27–40)*	***40 (32–47)***	*44 (32–57)*	*33 (28–37)*	*51 (45–57)*
*Overall Average*	*55 (51–59)*	*61 (55–68)*	***72 (69–75)***	*69 (64–75)*	*59 (53–65)*	***79 (75–82)***
	***N*** **= 4814**	***N*** **= 4822**	***N*** **= 4978**	***N*** **= 4813**	***N*** **= 4843**	***N*** **= 4836**
**Stillbirth rate, per thousand live births (95% C.I.)**	15 (6–15)	17 (10–23)	2 (0–5)	25 (9–40)	19 (14–23)	11 (8–14)
**Caesarean rate, % (95% C.I.)**	2.0 (0.2–3.8)	**4.8 (0.0–9.7)**	**7.6 (1.4–13.9)**	2.9 (0–5.7)	2.3 (0–4.8)	1.4 (0–3.9)
**Mothers’ referral rate, % (95% C.I.)**	0.1 (0.0–0.3)	0.7 (0.1–1.2)	0.1 (0.0–0.2)	0.4 (0.1–0.7)	0.5 (0.1–0.9)	0.2 (0.0–0.4)
**Newborn referral rate, % (95% C.I.)**	1.4 (0.0–2.8)	1.9 (0.8–3.0)	0.5 (0.0–1.2)	2.1 (1.0–3.2)	1.3 (0.6–2.0)	**0.5 (0.0–1.5)**

*bold means significant *p*-value < 0.05 when compared to first assessment results, *CS* case sheets assessed after discharge, *OBS* direct observations, *INT* interviews conducted about a day after birth

### Pause point 1 (during admission)

Conducting abdominal and vaginal examinations were high and improved (> 95%) further. Maternal blood pressure and foetal heart sound assessment improved from 52 to 83% in Group 1 and 67 to 97% in Group 2. The change in hand hygiene before the vaginal examination was slow initially. After introducing the alcohol-based hand rubs, it improved tremendously (16 to 73% in Group 1 and 49 to 90% in Group 2). Overall, the average performance during admission consistently improved over time, from 63 to 87% (p = < 0.001) in Group 1, and from 77 to 96% (*p* = 0.001) in Group 2. Among other indicators, monitoring the progress of labour (i.e. assessing the uterine contractions, foetal descent and foetal heart sounds at least twice) improved to above 50% in both groups.

### Pause point 2 (during childbirth by vaginal route)

The use of clean cord cut was almost universal throughout the study. Pre-filling the injection oxytocin significantly improved from 67 to 97% and 78 to 94% in Groups 1 and 2. But administering injection oxytocin within 5 minutes remained less than 80% in Group 1 while it improved from 83 to 94% in Group 2 over time due to sustained long-term mentoring. The availability of clean and warm towels improved from 47 to 76% in Group 1. Keeping the bag and mask ready dropped from 85 to 70% in Group 1 while it stayed above 90% in Group 2. The average performance during childbirth improved from 74 to 84% (*p* = 0.493) in Group 1, and from 86 to 92% (*p* = 0.017) in Group 2. Among other indicators, induction of labour reduced from 30 to 22% in Group 1 and significantly from 39 to 9% in Group 2. We also noted improvement in pre-heating the warmer, hand washing before delivery, delayed cord cut, mothers’ privacy and counselling for progression of labour (results not shown).

### Pause point 3 (within 1 hour of childbirth)

Drying the baby immediately was 85% or above through all three assessments. Within one hour breastfeeding improved significantly from 42 to 79% and 45 to 78%, and the assessment of the mother’s vitals significantly improved from 13 to 67% and 45 to 82% in Groups 1 and 2. The changes in measuring of baby’s weight and assessing the uterine tone were inconsistent; they ended at about 70% or above by the third assessment in both groups. The average performance of evidence-based practices immediately after childbirth improved from 57 to 76% (*p* = 0.001) in Group 1, and from 69 to 76% (*p* = 0.002) in Group 2**.**

### Pause point 4 (before discharge)

Measuring mothers’ temperature and counselling for family planning consistently improved in both groups, while counselling for newborn danger signs reduced significantly. The average performance at discharge improved from 24 to 40% (*p* = 0.070) in Group 1, and from 44 to 51% (*p* = 0.008) in Group 2.

### Overall performance

**Fig. 3:**
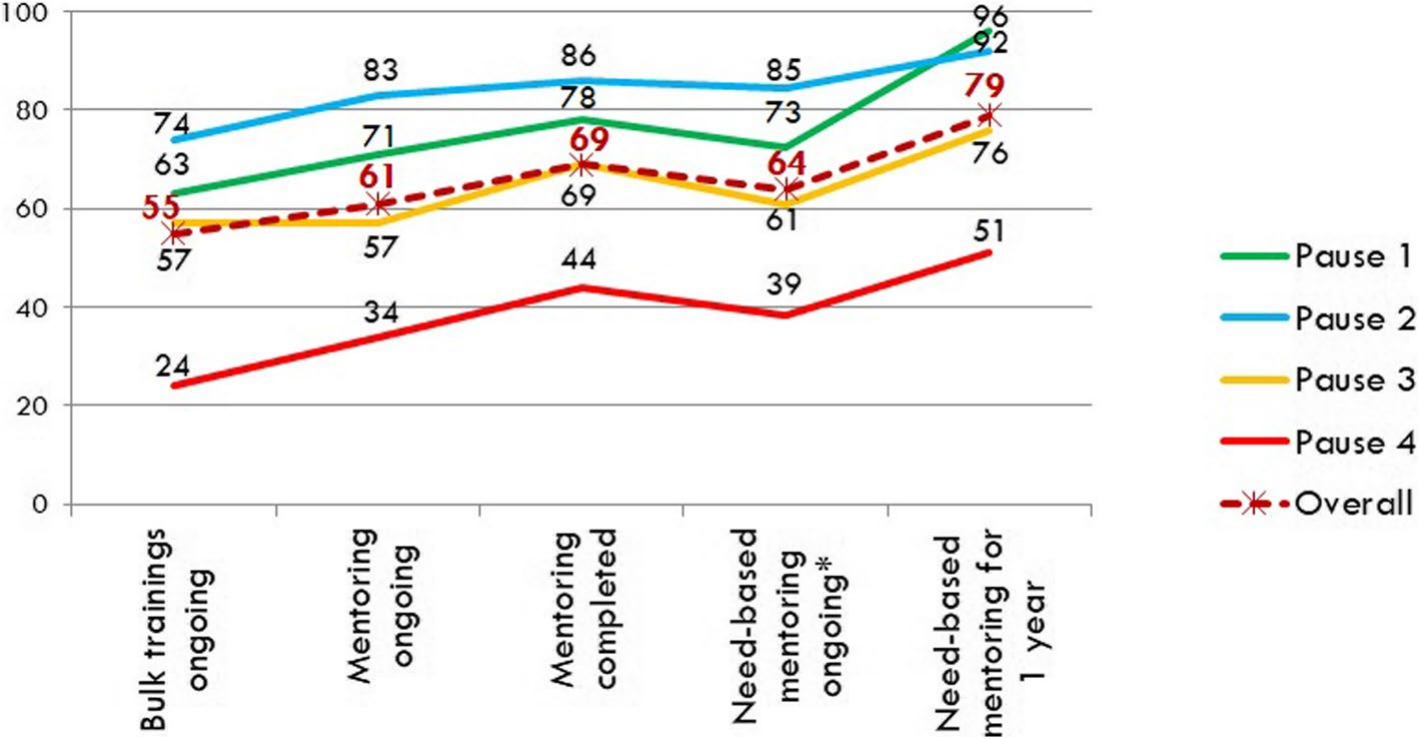
Average adherence to practices in pause points and overall concerning the stage of program implementation, % *average of Group 1 time3 and Group 2 time2

The overall average of the 20 scored practices, improved significantly in Group 1 to 72% and Group 2 to 79% (*p* < 0.001 for both). We additionally charted the average performance as per the program implementation status at any assessment, Fig. 3. There was a positive trend association towards improvement in quality of services across all pause-points and overall averages with continuous structured mentoring followed by need-based mentoring.

### Performance scores of facilities

**Fig. 4:**
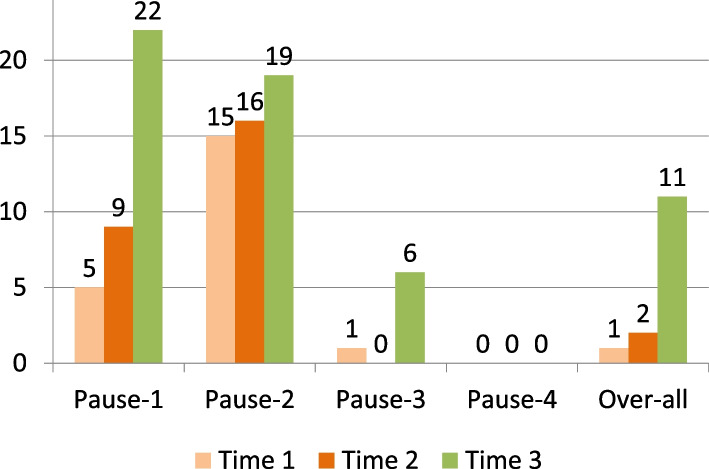
Number of study facilities with score of 4 or more in pause points, and overall score of 14 or more over time, *N* = 24

We scored facility performance and found that out of 24 hospitals, the performance improved to satisfactory levels in 22, 19, 6 and 0 hospitals for pause points 1, 2, 3 and 4 respectively, in only 11 hospitals overall (Fig. 4).

### Outcomes

Only 3% of case sheets classified any high-risk or complication in the diagnosis; we were not confident of the quality of reporting thus we could not make any quantitative assessments. However, we summarise the noted management of common complications in Additional Table 2. During direct observations of few complications, we noticed improved confidence in staff in stabilising the cases of PIH, managing PPH and resuscitating the child in asphyxia.

Among studied outcomes, the stillbirth rates reduced significantly (*p* < 0.001) across the groups over one and a half years: in Group 1 from 15 to 2 per 1000 live births and in Group 2 from 25 to 11 (Table 3). During this period the caesarean rates increased in Group 1 facilities but reduced in Group 2 while the mother referral rates remained steady. The newborn referrals to newborn care units significantly decreased in Group 2; the proportion of neonatal jaundice and birth asphyxia reduced, while hypothermia increased, and pre-term was similar over time. As we extracted data from registers, we noted fewer missing data on key indicators by the third assessment.

### Qualitative results

We present the qualitative results in three broad sections: 1) Effectiveness of intervention; 2) State’s ownership and program implementation, and; 3) Sustainability. Lastly, we summarise the facilitators and challenges and suggestions for the sustainability and scalability of the program. Table 4 lists stakeholder statements under common themes and sub-themes.

**Table 4:** Select quotes form the stakeholders’ interviews conducted from Barmer and Dholpur districts of Rajasthan

Theme/ Sub-themes	Illustrative Comments
**EFFECTIVENESS**	
Knowledge and Competencies	*-“There are changes in conducting delivery and methods of baby care which was not done prior to Dakshata, we were unaware about the processes and required care to be given during childbirth.”* Staff nurse *-“The process and practices of shifting patient from ANC to the ward and labour table, including usual and routine check-ups… the importance of these checkups was not known to us before Dakshata…In SBA (skilled birth attendance training) we were not told to handle in detail about high risk or complication for mother. In Dakshata we have been told very nicely about PPH, APH. In SBA there is no monitoring.”* Staff nurse *-“after Dakshata came, we came to know that hand wash should be done before wearing gloves and hand wash should be done after removing gloves”, Staff nurse* *-“Dakshata is ensuring that standard protocols are followed and staffs are well-trained....other MCH programs are not so specific, Dakshata is focused on Intra-Partum care.… Even after UNICEF program, there were not many changes which were possible after Dakshata..”* Principal Medical Officer (PMO)
Perception of Quality and service delivery	-**“** *More awareness and sensitization is seen among the service providers towards improving practices, in turn leads to providing quality services.”* PMO *-“After the delivery, all the instruments are dipped in the hypochlorite solution and the instruments are not used without sterilization.”* MO*.* *-“…through examination and investigation like BP, FHS, fundal height, HB, sugar we are able to find the risk in mother and the new born and if we are able to manage we do it or else we refer due to which the chance of death gets reduced..”* Staff nurse *-“Dakshata trains us and our staff how to deliver quality services with limited resources… It covers all aspects of care and services from registration to discharge, it also covers Emergency Obstetric Care…”* Medical Officer
Effectiveness	-“*..The golden minute after delivery of the baby and in case of any complication, we are doing all the mentioned practices according to what we have learned from Dakshata. In a way, this has reduced the chances of mother and baby death.”* Staff nurse *-“Now all the practices seem easier to us and it has become our habit to do them. We feel nice to do it for the benefit of the patient. After the patient comes, immediately we check their weight, BP, pulse, temperature, FHS which is also for our own benefit. This enables us to identify IUD, and high-risk mothers.”* Staff nurse *“we used to refer in case of PPH management but now we make use of balloon tamponade for its management.”* Staff nurse
Outcomes of *Dakshata* program	*-“…Now all the facility is available here and we provide baby care, they (clients) aren’t facing money loss and time waste (due to referral) as earlier…Still birth has also become less than before.*” Staff nurse *-“Due to the good services and decreased referral rate, patient load has increased and credit goes to Dakshata training.”* MO *-“MMR and NMR are reduced by improving labour room practices, neonatal complications also reduced.”* Chief Medical Health Officer (CMHO)
**PROGRAM IMPLEMENTATION AND STATES OWNERSHIP**	
Leadership	*-“I have realized that getting sanction from the district for the advanced instruments and machines will take long time. So I have requested some donors and got NIBP machines for critical patients, and pulse-oximetry.”* PMO *-“..Local body committee is formed at our CHC, asks the labour room staff and themselves to assess their own practices and mistake through internal assessment. This was possible due to the constant efforts of, JHPIEGO mentor.” MO*
Mentoring and Supervision visits and periodic assessment	*-“Reformation took place in our facility after Dakshata which includes infrastructural and supply related changes like installation of elbow taps, L shaped entrance from the Labour Room, Autoclave Drum* etc.*,…. change in attitude of our staff as well as change in practices is due to the practical mock-drills conducted under Dakshata.” PMO* *-“Mentoring and supervision visits (MSVs) have improved quality of care in labour room.” Staff nurse* *-“MSVs are responsible for improved accountability amongst staff.”* PMO *-“We are unable to give more time because we continuously work in OPD… mentoring once in a month can tell us the gap which can help in our improvement.”* MO *-“Microanalysis of labour room and charts and checklists are different in Dakshata comparing to other programs… Remarkable changes in last few years after Dakshata, as compared to 2010.”* CMHO
Motivation and encouragement	*-“… but now after getting the training everyone is doing it (service delivery as per protocol) because all these things come under routine and it doesn’t feel good if we don’t do it.” Staff nurse* *-“There’s lack of motivation amongst the staff because of no capitation method of payment like that in private sector. Fee for service motivates the staff to work harder which is why they (private hospitals) are better on Quality front.”* *-“To maintain accountability among the staff, I don’t believe giving them monetary incentives is a good option. But I have nominated the names of the good performers to the district and also on the occasion of Republic day and Independence day I awarded them with a memento and garland so that it will also motivate other staff.”* PMO
**SUSTAINABILITY**	
Improved infrastructure and demand supply chain	*-“After Dakshata everything has improved very much. Drug supply, instruments all A to Z is available… if there is a problem then PMO sir (facility leader) makes the purchase and brings it to us.”* Staff nurse. *-“There are many problems, no proper washing and drying area, not even place to wash baby towels. There is no changing area. No separate cleaning staff for labour room, shortage of staff for labour room. There is problem every where…”* Staff nurse
Improved competence and quality of service delivery	*-“..Now even if we get support of 1–2 staff we are able to provide proper services and care to the mothers and the newborns and follow practices mostly according to Dakshata protocols … … .we could see the good results, so we felt good and started performing it on a regular routine..”* *-* “*… … we will try that the same practice continues in the future, whoever comes for institutional delivery should get the proper service….”*
Continued skill upgradation	*-“We got trained completely in all aspects of Dakshata. If any new things come up in Dakshata, and if we are reoriented and upgraded from the Government’s side, then we will work accordingly … now it is embedded into our routine and with daily practices we are following Dakshata guidelines.”*
Mentoring and Periodic assessments	*-“In other programmes there is no supervision and follow up but in this program there is follow up, which help us to realize our mistakes and also scope to rectify them.”* Staff Nurse *-“MSV given by JHPIEGO is very useful, but I am skeptical about the future when the role would be fulfilled by a Government personnel as staff beleves follow-up by government is usually futile….. Government MSV will be limited to paper and would not be in any comparison with existing JHPIEGO mentors.”* PMO, MO
Motivation and accountability	*-“We have formed a local body committee at our facility level, consisting of OT in-charge, MO, Anesthetist, Pediatrician and Gynecologist and staff nurse. Their duty is to look after the quality of the labour room …”* MO, PMO *-“… local body committee asks the labour room staff and themselves to assess their own practices and mistakes. We conduct internal assessment. This was possible due to the constant efforts of JHPIEGO mentors.”* PMO
Link to other programs	*-“Monetary incentives to the mothers which they are getting from the programmes like JSY can reduce issues like Leaving against medical advice and in turn reduce MMR and NMR.”* MO
**CHALLENGES**	
Community level	*-“More of anaemic patients come here with 4 to 5 g Hb as there is no proper ANC so there could be more chances of PPH.”* PMO *-“… when mothers come for the delivery, they don’t want to wait for a longer duration and sometimes they want the delivery as soon as possible…”* Staff nurse *-“… Since the Free Drug Scheme has started, self-prescription has become common …”* MO
Shortage of staff and inadequate resources	*-“In such a vast district like Barmer, LR practices are highly burdened due to lack of manpower and inadequate infrastructure.”* CMHO *-“We face a demand-supply gap. When we raise a purchase of more number of drugs (like Vitamin-K), only a limited amount of drugs are delivered from the District Warehouse. We are struggling to establish pathways for procurement and sometimes are compelled to buy drugs like Oxytocin from market at a higher rate.”* PMO
Local political influence and interference	**-“** *Political influence and polarization impacts the program implementation. There is also discrimination among the staff on the basis of caste, power, role and gender. Sometimes medical officers cannot interfere in these issues, as they (service providers) get him transferred from there using their connections….” PMO,* Mentor
Unmotivated staff and reluctant doctors	*-“Doctors in DH don’t sit in OPD. To get excused from OPD they go for rounds of PN ward for about 3–4 hours. After OPD hours, they call the patients to visit them in their private clinics. Back in the DH, doctors are taking care of their respective patients who have visited them in their residents… Specialist doctors (O&G) and MOs are not attending the Dakshata training or mentoring, as they have assumed that it is meant only for the nursing staff.”* Mentor
Inconsistent support from the administration	*- “… … ..government doesn’t maintain consistency, fails to reinforce training, and depends largely on paper-work. They also do not show familiarity with staff and remain as an authoritative figure.”* PMO, MO

Table 4 Select quotes from the stakeholders’ interviews conducted in the Barmer and Dholpur districts of Rajasthan.

## Effectiveness of intervention

### Knowledge and competencies

Staff gained new knowledge about standards and protocols for delivery care and management of complications. The nurses reported that they felt more competent and confident in conducting low-risk vaginal deliveries by following the checklist and protocols. They were able to manage complications such as PPH and newborn resuscitation and provide stabilising care for complications with existing resources, before referral. They learned how to talk to the client and counsel them. During the second assessment, we observed a deeper understanding of the program and clinical management. Nurses were more elaborate about the comprehensive and structured way of providing care to the mother and the newborn, from admission through discharge and the necessity to perform all the required steps for prevention of complications to get desired outcomes. They reported that earlier it took them much longer and a lot of effort to practice all the recommended tasks. But with time, the tasks seemed easier, and the learnt skills were getting embedded in their routine, as per protocols, followed in a systematic manner.

### Resource modification

Staff noted considerable improvement in infrastructure, and availability of resources, including protocols, that motivated them and facilitated appropriate service delivery. The periodic assessments by the mentors helped them to identify the gaps and rectify them. Mentors helped in overcoming administrative procedures to avail the resources. They also helped in identifying and instituting local solutions for operational problems.

### Perception of quality and service delivery

Earlier the staff perceived quality in terms of infrastructure improvement, availability of relevant trays, sterilisation of instruments, use of gloves and hygiene practices. But after the program, they distinguished quality as improvement in the actual provision of appropriate clinical care and management of complications rather than infrastructure and supplies alone. As told by staff, the emphasis increased on measuring vitals of women, high-risk screening, measuring foetal heart sounds, effectively performing steps in childbirth, administering injection of oxytocin after childbirth, management of post-partum haemorrhage, and use of Magnisium sulphate MgSO4 for prevention and management of eclampsia. By the second program assessment, the staff also emphasised infection control. The nurses mentioned that sterilisation of equipment and use of hypochlorite solution was not done before the *Dakshata* program. Hand hygiene was mentioned as one of the practices difficult to follow, especially when the load was high. With the introduction of hand rubs, there was some improvement in hand hygiene. The participants accepted that they had stopped unnecessary induction of labour, multiple PV examinations and inappropriate management such as fundal pressure. They also added counselling to the mothers and attendants before discharge as components of quality service delivery.

### Effectiveness

Effectiveness of the program was told to be in being able to provide care as per the need of the pregnant women and reducing adverse outcomes. Over time, the staff put more emphasis on physical assessment at the time of admission, filling of partograph, counseling the mother and birth attendant for early identification and tracking of the high-risk cases. The staff talked about the concept of golden minute, newborn care, and resuscitation. A nurse mentioned using balloon tamponade before referring a PPH case which she never did in the past which was also part of training for post-partum hemorrhage management. They also learned and practiced good behaviour with the client.

### Outcome of Dakshata program

The stakeholders believed that improved care as per *Dakshata* program protocols had led to improvement in quality of care and service, reformation in infrastructure and supply, efficient management of complications, reduced referrals, reduced stillbirth, maternal and neonatal deaths. There was an improvement in nurses’ role in decision-making for client care and labour room maintenance.

### Accountability

We noted a positive change in the service providers’ attitudes toward providing quality services with a clearer understanding of the benefits associated. The staff believed that the mentoring and periodic assessments contributed to the service providers being aware and proactive towards their duties, with an increased sense of responsibility.

## State’s ownership and program implementation

We reviewed the facilitators, challenges; sustainability, and scalability of program components.

### Ownership and engagement by state health department

The state health department completely owned the implementation of the program and led it with support from the external agency. The national-level stature of the program drove motivated implementation. Rajasthan was among the first few states to successfully implement the program and much appreciated too. State regularly monitored the program activities and performance, undertook timely decisions to fill in the gaps, provided administrative support and resources, and conducted repeated sensitisation and training for lagging practices. We also noted that after our periodic feedback from assessments, the state took appropriate measures to improve services. The state realized that the success of the program needed to be sustained and improvement should continue. Thus, the state recruited 17 district mentors, and deputed in-service block mentors (nurses/doctors), to continue the program after external support was phased out.

### Leadership

The district mentors appreciated the state leadership and attributed the success of the program implementation to the state government health officers. The commitment and accountability at the state-level trickled to the lower-level administrators, hospitals, and service providers too. Some hospital administrators were very proactively involved in bringing changes through the *Dakshata* program. Some other fulfilled the essential resource requirements through public-private partnerships or donations. On the other hand, a few interviewees mentioned poor leadership at the district and hospital, and the staff struggled to perform well due to inadequate support.

### Monitoring

The state implemented a structured monitoring mechanism, later also strengthened by a newly designed android-based software application. The application helped mentors in conducting on-spot periodic assessments, scoring the performance, and giving feedback to the staff or in-charges. It also helped the district and state administrators to track real-time the hospitals’ performance and mentors’ visits. The district administration in Barmer also periodically reviewed the program in district maternal and child health review meetings.

### Efficiency of training, mentoring and periodic assessments by partner agency

The hospital administrators and service providers appreciated the short duration and effective mode of training. The *Dakshata* training was perceived to be different from previous training, including that for skilled birth attendance. Mentoring and supervision visits and periodic assessments were perceived to be the most prominent feature and strong pillars of the *Dakshata* program. The staff mentioned that mentoring visits helped them internalize the standard practices without having to put a lot of effort into the learning process. The mock drills and the briefing-debriefing exercises helped in improving clinical skills. All the stakeholders admitted that mentoring in the staff’s workplace was more conducive and regular follow-up improved the sense of responsibility and accountability at all levels. Mentoring visits were of particular importance in setting all the infrastructure and resources right.

### Motivation, encouragement, awards

The state provided recognition and award (mementos, certificates) to the best performers. A few providers and administrators felt that these measures encouraged and motivated staff and improved accountability. The review of the program in monthly maternal health meetings at the district and state levels also provided opportunities for cross-learning and finding solutions for common challenges.

## Sustainability

The stakeholders shared their understanding of factors for the successful sustainability of the program, as well as the threats.

### Improved infrastructure and demand supply chain

The labour room infrastructure and demand supply chain significantly improved and were among the key factors for improvement in staff motivation and service delivery. The mentors played the central role in facilitating these changes, and post phase-out the labour room in charge would need to pro-actively take over this role to maintain resources.

### Improved competence and quality of service delivery

The evidence-based practices improved, and staff noted the difference in their practices pre- and post-*Dakshata*. The program encouraged and enabled them to improve the quality of services, and these improvements gave them job satisfaction. They aimed to continue the same practices and improve further with guidance.

### Continuous skill upgradation

The stakeholders mentioned that regular skill upgradation training or mentoring were required to sustain quality services and incorporate newer evidence-based improvements to clinical care.

### Quality of mentors

All the stakeholders stated dynamic relationship with JHPIEGO mentors; mentoring and periodic assessments made the program much desired. These JHPIEGO mentors were mostly MBBS doctors, while the new government mentors were a mix of trained para-medical staff and nurses and very few doctors. Administrators told that post-phase out, the government mentors have to do a lot to fill in the shoes of earlier mentors. The service providers were skeptical about the performance after shifting from JHPIEGO mentors to government mentors.

### Motivation and accountability

The recognition and monetary awards were motivation to continue good work.

### Linkage and support from other programs

The providers told that the incentives provided under *Janani Suraksha Yojana* ensured that the mother stayed at the hospital for at least 48 hours post-delivery, which provided an opportunity to deliver post-natal care. Programs such as *Kayakalp* helped in instituting sanitation and hygiene; *Laqshya* had standards for resources and services and provided certification of quality of services in labour rooms and pediatric units.

## Challenges and suggestions for effectiveness, accountability and sustainability of the *Dakshata* program

### Community level care and practices

Inadequate antenatal care, high prevalence of severe anaemia, late arrival at the health facility, poor awareness about delivery processes, unhygienic practices, and requests for early intervention or early discharge negatively affected the quality of services. Staff reported referring most of the late arriving complication cases.

### Shortage of staff and inadequate resources

amidst high patient load worsened the ability to provide quality services and documentation; and postnatal care was negligible. The state recruitment for 800 nurses was on hold for 3 years due to a court litigation. Despite huge improvements, the gaps in infrastructure and supply chain were still pertinent.

### Local political influence and interference

District-level officials were displeased with political interference that resulted in the irrational positioning of humans and other resources. Some service providers used political influence as a means to escape from their daily duties and responsibilities; they were difficult to supervise or discipline.

### Unmotivated staff and reluctant doctors

Not all staff were similarly motivated. Most obstetricians and a few medical doctors did not want to participate in the *Dakshata* program. In their understanding, the program was only for nurses and support staff.

### Malpractices

There were certain reports of malpractices, such as doctors referring the patient to his/her private clinic, and staff asking for monetary remuneration for otherwise free services.

### Inconsistent support from the administration

District and hospital participants reported inconsistent support from the government for adequate human resources, reinforcement of training, and monitoring. Staff perceived that the government had a poor understanding of the practical problems at the bottom level. Staff felt that the Government would not be able to provide proper follow-up mentoring after the phase-out of JHPIEGO mentors.

### Suggestions

We also asked the stakeholders about the suggestions on how to improve the quality of obstetric care and for *Dakshata* implementation program. The suggestions are compiled below in brief.

1. Provide pre-service training and regular refresher training in a systematic manner close to the workplace and update the latest protocols.
2. Establish stringent monitoring and feedback system; strengthen periodic assessments through mentors and reviews by administrators in routine MCH meetings.
3. Empower and support the new government mentors for efficient mentoring and periodic assessments. Monitor mentoring.
4. Ensure minimum human resources as per the load, particularly nurses. Do not rotate staff; commit trained obstetric staff to only obstetric wards or labour rooms.
5. Ensure availability of essential equipment and supplies; maintain consistency.
6. Adopt a quality improvement approach in line with programs such as *Laqshya* and *Kayakalp*; make use of these for the identification of local problems and solutions. Formation of quality circles/ teams supported by the administration may help.
7. Establish advisory support and redressal mechanism for technical as well as managerial concerns.
8. Strengthen and improve antenatal care and empower the community regarding obstetric care. Target specifically poor performing and deprived communities.

## Discussion

In this evaluation of a complex intervention instituted in a public health system of a state with comparatively lower resources and poorer maternal and newborn indicators among states in India [16, 17], we noted significant improvements in the quality of care provided and acceptability of the Dakshata program by staff and implementers. We adopted the most suitable evaluation design in the provided time frame, ensuring that we cover all the stages of the intervention to understand short-term as well as mid-term changes.

Overall, of the studied 20 key practices, four were at > 90%, six at 80–90%, seven at 50–80%, and three less than 50%. The evidence from other states in India also suggests that not all the practices were adopted with the same rigour (pause points 2 and 3 were adapted better), and sustainability of the improved practices was challenging [8, 10, 11, 15, 27]. In our evaluation, a few practices from pause points 1, 2 and 3 were adopted quickly after the program. There seemed to be a felt need for knowledge and skills and a systematic approach to identifying and managing complications [4]. The program also empowered nurses and facilitated better teamwork and support from the doctors, which contributed to decision-making and better service delivery by nurses [4, 9, 28]. Many other practices improved slowly with continuous reinforcement and by ensuring adequate resources [2, 4, 28]. On the other hand, a few practices were resistant to change due to personal or health system issues [29]. These were mostly pertaining to post-partum and postnatal care [8, 10] where the staff did not directly feel responsible for post-hospital outcomes. Hand hygiene and postnatal counselling were amongst the difficult practices to improve; similar to other evaluations of quality improvement for intrapartum care [9, 10, 27]. As the adoption of evidence-based practices happens at its own pace, for an effective mentoring program, it is important to understand the staff’s priorities, assigned importance to certain practices, the resistance, and existing national standards and protocols; and plan for a long term adaptable mentoring curriculum [6].

Among the two large trials in India, in the BetterBirth trial of Uttar Pradesh, there was a sustained improvement during the 8 months of coaching but noted a drop after 4 months since the coaching ceased; there was no change in maternal or neonatal mortality [8]. We also noted in *Dakshata* the tendency for adherence to regress over time. In our assessment in Rajasthan, mentoring continued for over one-and-half to 2 years which improved or sustained certain practices led to higher caesarean section rates and a higher ratio of referrals in Group 1 representing the initial program stages. With increased resources and confidence gained after the sustained program, the referrals could decrease and stabilise as in Group 2 representing advanced program stages. The stillbirth rates decreased in both the groups; and were close to the state average of 6/1000 live births [17]. Global evidence on the use of WHO SCC shows a mixed impact on maternal or perinatal mortality [7, 8, 12, 13, 29].

The technical partner adopted a scientific approach to pilot testing and scaling up, using an adaptive and iterative prototyping approach and sharing information and feedback with key stakeholders [30]. The key to *Dakshata’s* success was client-oriented and service provider-centric mentoring complemented by periodic assessments by the technical partner which is reproducible by government mentors. The qualification and capabilities of the mentors, and the support and resources provided to them, especially in a hierarchy-driven environment such as in India, can be the determinants of the success or failure of the program [6]. The support of hospital leadership [6, 31, 32], adequate resources, and availability of staff at the time of mentoring are essential for successful mentoring. *Dakshata* program, unlike other SCC-based interventions, ensured better preparedness and resource availability before initiating capacity building. Frequent transfers and rotation of staff posed a necessity for repeat training and a continuous mentoring program [6, 33]. The program requires greater emphasis on the effective use of data from periodic assessments. Any mentoring or coaching program would require strong commitment; pro-active monitoring of the mentors; feedback and supportive management from the district and state administration [6, 9, 18, 20]. The state of Rajasthan was highly committed and involved in the planning, implementation, and monitoring of the program, and was the mainstay of its success. A similar commitment and effort shall have to be continued to further improve the practices [6]. For sustaining the current mentoring model, the state requires innovative solutions to embed mentoring culture within the health system; and devise local, programmatic and policy solutions for integrated quality improvement initiatives and programs in the country [1, 3].

The methods considerations and limitations are discussed in detail elsewhere (submitted); however, a few key limitations are worth noting. Some important indicators could not be included as we were not confident of their documentation. There is the possibility of inter-observer bias although we provided repeated training and supervision. There could be the Hawthorne effect [34] but non-participatory observations in substantial numbers were the best methodology for assessing practices. We interviewed postnatal women after 24 hours of birth to be able to capture most post-natal women who are otherwise likely to leave early against medical advice. By doing so, we were likely to under-report pause 4 practices on counselling that were to be provided at the time of discharge under the program. Evaluation with controls would have been a better study design; however, all the districts had some *Dakshata*-related intervention underway when the evaluation commenced. Another observation after phase-out would have provided better evidence on sustainability.

## Conclusion

The adherence to practices recommended under the *Dakshata* program improved to high levels in admission and just before birth (pause point 1 and 2), improved to moderate levels in the first hour postpartum (pause point 3) but stayed poor at faciality discharge (pause point 4) even after one to two years of mentoring. Importantly, the stillbirth rates reduced significantly in both groups over 18 months, significantly better in Group 1 than Group 2. The structured mentoring followed by need-based mentoring with monitoring and administrative support from the state was a successful implementation model. The intensive support from a technical partner (Jhpiego) was essential for resource-constrained states to give them a strong head start. For long-term sustainability, policies are required for building mentoring culture and integrating quality improvement programs.

## Supplementary Information

**Additional file 1:** **Supplementary Table 1.** Human resource, protocols, hygiene supplies, essential trays in study hospitals, over time.

**Additional file 2:** **Supplementary Table 2.** Management of complications during childbirth in the study hospitals.

## Data Availability

Most of the data and findings from the evaluation are shared in this manuscript and the supplementary file. The datasets generated and/or analysed during the current study are not available in the public domain due to insufficient funds for uploading on the Institute’s data repository, but the data sets are available from the corresponding author upon reasonable request.

## Abbreviations

ANMS: Auxillary Nurse Midwife
JHPIEGO: John Hopkins Program for International Education in Gynaecology and Obstetrics
MMR: Maternal Mortality Ratio
NMR: Neonatal Mortality Ratio
SCC: Safe Childbirth Checklist
WHO-SCC: World Health Organisation Safe Childbirth Checklist
