# Supplementary material for: Evaluation of Dakshata, a scale-up WHO SCC and mentoring-based program, for improving quality of intrapartum care in public sector in Rajasthan, India: repeated mixed-methods surveys
Source: Arch Public Health. 2023 Apr 18;81:57. doi: 10.1186/s13690-023-01028-z (PMC10111820; doi:10.1186/s13690-023-01028-z)
Supplement: Supplementary file 1 — Additional file 1: Supplementary Table 1. Human resource, protocols, hygiene supplies, essential trays in study hospitals, over time. [file 13690_2023_1028_MOESM1_ESM.docx]

Supplementary Table 1: Human resource, protocols, hygiene supplies, essential trays in study hospitals, over time

|  | **Group 1, N=12** | | | **Group 2, N=12** | | |
| --- | --- | --- | --- | --- | --- | --- |
|  | ***Bulk trainings ongoing*** | ***Mentoring ongoing*** | ***Need based mentoring ongoing*** | ***Mentoring completed*** | ***Need based mentoring ongoing**** | ***Need based mentoring for a year*** |
| **Deliveries per month, Median (IQR)** | | | | | | |
| - District hospital/MCH hospital | 809 | 899 | 1027 | 493 | 546 | 570 |
| - Sub-district hospital | 247  (223-272) | 247  (118-377) | 193  (94-292) | 224  (222-275) | 191  (154-198) | 150  (93-238) |
| - CHC/PHC | 158  (117-234) | 130  (113-159) | 88  (62-141) | 103  (97-130) | 86  (69-100) | 78  (68-96) |
| **Human Resource in labour room, Median (IQR)** | | | | | | |
| Number of obstetricians | 1 (0-2) | 1 (0-2) | 1 (0-2) | 1 (0-2) | 1 (0-2) | 1 (0-2) |
| - Trained in *Dakshata* | 1 (0-2) | 1 (0-2) | 1 (0-2) | 1 (0-2) | 1 (0-2) | 0 |
| Number of nurses | 4 (3-6) | 4 (3-6) | 5 (4-9) | 6 (4-8) | 6 (4-8) | 6 (4-7) |
| - Trained in *Dakshata* | 3 (2-4) | 4 (3-5) | 5 (4-9) | 5 (2-8) | 5 (4-5) | 6 (4-7) |
| All day functional Caesarean OT | 4 | 4 | 4 | 5 | 5 | 6 |
| **Availability of protocols, n** | | | | | | |
| - Skill Birth Attendance | 5 | 4 | 5 | 4 | 3 | 6 |
| - Using Partograph | 7 | 7 | 12 | 9 | 6 | 11 |
| - Active Management of 3^rd^ Stage of Labour | 10 | 8 | 12 | 10 | 8 | 12 |
| - Using ante-natal corticosteroid | 6 | 5 | 8 | 2 | 3 | 4 |
| - Preterm labour | 3 | 3 | 8 | 6 | 2 | 1 |
| - PPH Management | 2 | 4 | 6 | 4 | 4 | 11 |
| - Eclampsia Management | 2 | 3 | 10 | 3 | 2 | 9 |
| **Essentials to maintain hygiene, n** | | | | | | |
| - Autoclave | 11 | 11 | 10 | 12 | 12 | 12 |
| - Water in labour room | 11 | 12 | 12 | 10 | 11 | 12 |
| - Attached hand-washing area, elbow operated tap | 11 | 11 | 12 | 10 | 12 | 12 |
| - Hand washing supplies** | 3 | 11 | 11 | 9 | 10 | 12 |
| - Colour coded bags to dispose biomedical waste | 9 | 12 | 12 | 10 | 10 | 11 |
| - Puncture proof container/disposal container | 6 | 10 | 12 | 9 | 5 | 9 |
| **Essential trays, n** |  |  |  |  |  |  |
| - Sterilised delivery tray | 6 | 8 | 10 | 9 | 5 | 5 |
| - Episiotomy tray | 9 | 10 | 10 | 9 | 8 | 8 |
| - Emergency drugs tray | 6 | 8 | 11 | 10 | 4 | 8 |

*a few facilities were undergoing renovation which disrupted resources and a few practices.
