# Supplementary material for: Evaluation of Dakshata, a scale-up WHO SCC and mentoring-based program, for improving quality of intrapartum care in public sector in Rajasthan, India: repeated mixed-methods surveys
Source: Arch Public Health. 2023 Apr 18;81:57. doi: 10.1186/s13690-023-01028-z (PMC10111820; doi:10.1186/s13690-023-01028-z)
Supplement: Supplementary file 2 — Additional file 2: Supplementary Table 2. Management of complications during childbirth in the study hospitals. [file 13690_2023_1028_MOESM2_ESM.docx]

Supplementary table 2: Management of complications during childbirth in the study hospitals

*(Information extracted from case-sheets for both vaginal and caesarean deliveries, supported by direct observations)*

|  | Time 1, (N=1,230) | Time 3, (N=1,232) |
| --- | --- | --- |
| **Classified as high-risk** | 41 (3.3%) | 43 (2.8%) |
| **Pre-term birth** | 43 (2.8%) | 23 (1.9%) |
| Any corticosteroid dose recorded | *9 of 43* | *4 of 23* |
| Remarks | Poor documentation, no protocols | ? Preterm referred / poor recording |
| **Antibiotic use** |  |  |
| Antibiotic required | 34 (3%) | 22 (1.8%) |
| Any antibiotic recorded | *11 of 34* | *2 of 22* |
| Additional use of antibiotic for those where eligibility not mentioned | 33 (2.2%) | 8 (0.7%) |
| Remarks | -Poor documentation, under-reporting | -Poor documentation, under-reporting |
| **Anaemia/ HB <8gm%** | 81 (6.6%) | 53 (4.3%) |
| Blood transfusion/ inj. iron sucrose | 2 severe anaemia cases; One case referred; Injection Iron sucrose started for the other post-delivery | 2 severe anaemia cases; management details not mentioned |
| Remarks | Haemoglobin levels mentioned in 76% case sheets | **Haemoglobin levels mentioned in 92% case sheets** |
| **Pregnancy induced hypertension** | 5 (0.3%) | 2 (0.1%) |
| Monitoring of blood pressure | 0-2 intermittent readings | 0-3 intermittent readings |
| Urine albumin | No mention | No mention |
| Treatment | 2 of 5 mention MgSO4, no mention of anti-hypertensives | 2 of 2 mention MgSO4 and anti-hypertensives |
| Remarks | - BP reading mentioned in 79%  -46 (3.7%) mentioned high BP but only 5 diagnosed as PIH/High BP.  - Tendency to not mention diagnosis if case stabilized  -Several PIH cases referred prior to admissions | **-BP reading mentioned in 94%**  -11 (0.9%) noted high BP of which only 2 diagnosed as PIH/High BP  - Tendency to not mention diagnosis if case stabilized  - Several PIH cases referred prior to admissions |
| **Post-partum haemorrhage** | 2 | 2 (1 vaginal, 1 caesarean) |
| Treatment | Injection Methergin/ Oxytocin administered, no mention of bimanual compression or transfusion. We observed one of these-bimanual removal of retained products | Injection Oxytocin administered, no mention of blood transfusion. Managed at the facility. |
| Monitoring vitals | No mention of intermittent pulse rate and blood pressure monitoring | Pulse rate and blood pressure monitoring done |
| Remarks | -Additional 13 cases were observed but diagnosis was not mentioned on the case sheets even later. 4 had perineal tear.  - Manual removal of retained products of placenta, injection Etamsylate and injection Oxytocin were used in most.  -3 received blood transfusion and one referred. | -Additional 4 cases observed. 3 mild haemorrhage cases managed by oxytocin; one retained placenta that was referred. |
| **Birth asphyxia** |  |  |
| Case sheet | -No mention of APGAR, Baby crying status mentioned in 90%;  -33 (2.7%) noted resuscitated | -No mention of APGAR, Baby crying status mentioned in 94%;  -58 (4.7%) noted resuscitated |
| Observations in labour room | -64 (14.7%) of 436 newborns had difficult or no breathing  -50 stabilised; 12 referred to SNCU; 2 died | -56 (11.8%) of 474 newborns had difficult or no breathing  -52 stabilised; 4 referred to SNCU |
| Remarks | - most newborns started crying on suction; staff less confident, response delayed for many | - staff confident, most stabilized in labour room, only a very small number required referral |
